# Thrombolytic therapy based on lyophilized platelet-derived nanocarriers for ischemic stroke

**DOI:** 10.1186/s12951-023-02206-5

**Published:** 2024-01-03

**Authors:** Martina Migliavacca, Clara Correa-Paz, María Pérez-Mato, Patrick-Brian Bielawski, Issan Zhang, Pauline Marie, Pablo Hervella, Marina Rubio, Dusica Maysinger, Denis Vivien, Pablo del Pino, Beatriz Pelaz, Ester Polo, Francisco Campos

**Affiliations:** 1 Present Address: Center for Research in Biological Chemistry and Molecular Materials (CiQUS), University of Santiago de Compostela, 15705 Santiago de Compostela, Spain; 2 Translational Stroke Laboratory Group (TREAT), Clinical Neurosciences Research Laboratory (LINC), Health Research Institute of Santiago de Compostela (IDIS), 15706 Santiago de Compostela, Spain; 3 Department of Pharmacology and Therapeutics, McGill University, Montreal, QC H3G 1Y6 Canada; 4 UMR-S U1237, Physiopathology and Imaging of Neurological Disorders (PhIND), Normandie University, UNICAEN, INSERM, GIP Cyceron, Institute Blood and Brain @ Caen-Normandie (BB@C), 14000 Caen, France; 5 Neuroimaging and Biotechnology Laboratory (NOBEL), Clinical Neurosciences Research Laboratory (LINC), Health Research Institute of Santiago de Compostela (IDIS), 15706 Santiago de Compostela, Spain; 6 Department of Clinical Research, Caen Normandie University Hospital, Caen, France

## Abstract

**Background:**

Intravenous administration of fibrinolytic drugs, such as recombinant tissue plasminogen activator (rtPA) is the standard treatment of acute thrombotic diseases. However, current fibrinolytics exhibit limited clinical efficacy because of their short plasma half-lives and risk of hemorrhagic transformations. Platelet membrane-based nanocarriers have received increasing attention for ischemic stroke therapies, as they have natural thrombus-targeting activity, can prolong half-life of the fibrinolytic therapy, and reduce side effects. In this study we have gone further in developing platelet-derived nanocarriers (defined as cellsomes) to encapsulate and protect rtPA from degradation. Following lyophilization and characterization, their formulation properties, biocompatibility, therapeutic effect, and risk of hemorrhages were later investigated in a thromboembolic model of stroke in mice.

**Results:**

Cellsomes of 200 nm size and loaded with rtPA were generated from membrane fragments of human platelets. The lyophilization process did not influence the nanocarrier size distribution, morphology, and colloidal stability conferring particle preservation and long-term storage. Encapsulated rtPA in cellsomes and administered as a single bolus showed to be as effective as a continuous clinical perfusion of free rtPA at equal concentration, without increasing the risk of hemorrhagic transformations or provoking an inflammatory response.

**Conclusions:**

This study provides evidence for the safe and effective use of lyophilized biomimetic platelet-derived nanomedicine for precise thrombolytic treatment of acute ischemic stroke. In addition, this new nanoformulation could simplify the clinical use of rtPA as a single bolus, being easier and less time-consuming in an emergency setting than a treatment perfusion, particularly in stroke patients. We have successfully addressed one of the main barriers to drug application and commercialization, the long-term storage of nanomedicines, overcoming the potential chemical and physical instabilities of nanomedicines when stored in an aqueous buffer.

**Supplementary Information:**

The online version contains supplementary material available at 10.1186/s12951-023-02206-5.

## Background

Ischemic stroke, caused by a clot occlusion of the cerebral blood flow (CBF), represents one of the main causes of disability and death globally. Nowadays, reperfusion strategies (pharmacological thrombolysis and mechanical thrombectomy), are the main approaches focused on the restoration of CBF [1]. Pharmacological therapy, based on the use of the recombinant tissue plasminogen activator (rtPA), is the only drug treatment for acute ischemic stroke. This recombinant enzyme converts plasminogen into plasmin, which degrades the fibrin network that composes the thrombus. In use from 1995, this treatment has been effective in the improvement of clinical outcomes, reducing disability, and achieving successful recanalization in approximately 30% of patients [2]. However, rtPA has many drawbacks that limit its use, such as: (1) a short half-life (4 min) which implies an administration performed as an initial bolus followed by continuous infusion; (2) neurotoxic side effects; and (3) risk of hemorrhagic transformation (HT). These limitations result in a short therapeutic window of 4.5 h from stroke, and subsequently, reduce the number of patients that can benefit from this therapy [3].

Mechanical thrombectomy has improved recanalization rates (to 80%) and decreased the risk of HT [4]. However, this strategy is useful when thrombi are located in large vessels. In fact, in some cases, rtPA is administered in combination with mechanical thrombectomy to increase recanalization rates and restore microcirculation [5, 6]. Despite these limitations, pharmacological thrombolysis remains an indispensable treatment in clinical practice [7].

Nanotechnology has emerged as a promising tool to overcome side effects and improve the efficacy of drugs, including thrombolytic agents. For instance, drug encapsulation has allowed a decrease in the therapeutic dose of rtPA, extend its half-life, reduce the risk of HT, target the effect to the clot region, and allow for controlled release [8–10].

In this field, the interest in biomimetic nanoparticles, based on the use of cell membrane-derived nanocarriers, has increased in the last years due to their multiple advantages; high biocompatibility, a cell membrane coating that can camouflage particles from the phagocytic system, and the intrinsic ability of membrane for specific targeting [11, 12]. Cell membrane-derived nanocarriers have been fabricated from different cell types including neural stem cells, macrophages, red blood cells, and platelets [12–20].

In the particular case of platelet membrane-based nanocarriers, this design is especially promising for improving the efficacy of thrombolytic stroke therapies as they have natural thrombus-targeting activity due to the presence of immunoregulatory proteins, such as, integrins (αIIbβ3, CD47), glycoproteins that functions as a receptor for von Willebrand factor (CD42b/GPIbα or GPIIb/IIIa), and other adhesion molecules (such as P-selectin, GPVI and cadherins), which interact with damaged vascular endothelial cells and fibrin. Furthermore, the outer platelet membrane decreases nanoparticle uptake by the mononuclear phagocyte system leading to a prolonged half-life of rtPA [21, 22]. Platelets are also small (2–4 μm) anucleate cells that simplify the process of membrane isolation, purification, and nanoparticle synthesis. Moreover, platelets are commonly transfused to patients that increase the biocompatibility and translational value of this type of nano-formulations [23].

The therapeutic effect of platelet membrane-derived nanocarriers for rtPA encapsulation have been well-demonstrated for stroke pathology [12]. However, critical parameters for future clinical applications, such as particle colloidal stability, aggregation, and storage have been poorly explored. Nanoparticles are often produced in an aqueous medium which increases the risk of particle aggregation during long periods of storage [24]. rtPA is also an unstable drug that is commercially supplied as a lyophilized powder and must be used within the first 6–8 h after reconstitution, per manufacturer instructions [25]. To overcome these limitations, lyophilization, also known as freeze-drying method, is frequently used to increase the shelf-life of nanomedicine formulations, facilitating their handling and storage [24].

In the present study, we have developed platelet-derived nanocarriers (defined as cellsomes or CSMs) to encapsulate rtPA (named as CSM@rtPA). Due to the stability of rtPA over time is a critical parameter for the nanoformulation fabrication process; after synthesizing and characterizing the CSM@rtPA samples, we have shown that lyophilization process maintains rtPA encapsulation and activity while preserving particle colloidal stability and size distribution. Moreover, the expression of platelet receptors in the membrane and the thrombolytic efficacy in vitro were studied. Finally, the therapeutic effect and the risk of hemorrhages following lyophilized CSM@rtPA administration were investigated in a thromboembolic model of stroke in mice.

## Results

### Synthesis and characterization of CSM@rtPA

**Fig. 1:**
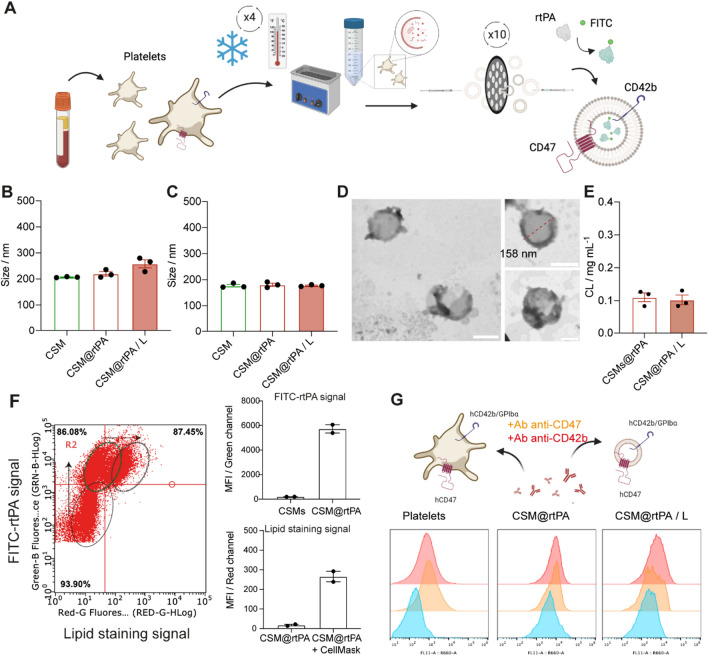
**A** Schematic representation of the preparation of rtPA-loaded CSM derived from platelets. **B** Representative mean hydrodynamic diameter of CSM and CSM@rtPA before and after lyophilization (CSM@rtPA/L) process measured by DLS. Data represent mean ± SEM (n = 3, independent samples). **C** Representative mean diameter of CSM and CSM@rtPA before and after lyophilization process (CSM@rtPA/L) measured by NTA. Data represent mean ± SEM (n = 3, independent samples). **D** Representative STEM-in SEM images of CSM@rtPA negatively stained with uranyl acetate. Scale bars: 200 nm. **E** Loading capacity of rtPA encapsulated in CSM samples. Data represent mean ± SEM (n = 3, independent samples). **F** Representative density plots and quantitative analysis of CSM and CSM@rtPA measured by FC. Scatter density plots of Green fluorescence signal (Green-B channel, rtPA channel) versus Red fluorescence signal (Red-R channel, CellMask DeepRed channel) for CSM, CSM@rtPA, and CSM@rtPA sample after labeling with CellMask Deep Red for lipid staining. Mean fluorescence intensities (MFI) for Green-B channel (**D**) and Red-R channel. Data represent mean ± SEM (n = 3, independent samples). **G**. Schematic representation of platelets and platelet-derived CSM surface proteins studied by APC-fluorescently labeled antibodies: anti-hCD47 Ab and anti-hCD42b/GPlba Ab. In vitro Ab binding to platelets and CSM@rtPA before and after lyophilization process. Representative MFI histogram of Red-R channel (APC signal) for platelets, CSM@rtPA and CSM@rtPA/L samples after incubation with APC-anti-CD47 and APC-anti-CD42b/GPIbα antibodies

We synthesized ~ 200 nm-sized CSMs vesicles derived from membrane fragments of platelets obtained by cell lysis following the protocol described in the literature [26]. Briefly, a liquid nitrogen-based freeze thaw process follows by an extrusion process was applied to engineer CSM nanocarriers preserving platelet surface proteins and markers involved in thrombus targeting. Then, CSM nanocarriers were loaded with FITC fluorescently labeled rtPA by the extrusion method (Fig. 1A), and after purification of non-encapsulated rtPA, samples were lyophilized and stored at − 20 °C.

Physico-chemical characterization of CSM, and CSM@rtPA before and after lyophilization (CSM@rtPA/L) revealed no significant changes among the sample monodispersed size distributions. Hydrodynamic diameters of colloidal CSM and CSM@rtPA dispersions in phosphate buffer saline (PBS) were recorded by dynamic light scattering (DLS) analysis, showing equivalent results between the three samples (mean hydrodynamic size in diameter of 225.0 ± 2 nm, 234.5 ± 1 nm, and 261.2 ± 28 nm for CSM, CSM@rtPA and CSM@rtPA/L respectively, Fig. 1B, Additional file 1: Fig. S1 and Table S1). Note, that the surface charge of CSM also remains constant throughout the different steps of synthetic procedure. Comparable ζ-potential values (~ 16 mV) are reported for CSM, as well as for fresh and lyophilized CSM@rtPA samples (Additional file 1: Table S1). Then single particle size evaluation reported by nanoparticle tracking analysis (NTA) (average core diameter of 182.8 ± 5.4 nm, 184.4 ± 11.6 nm, 187.3 ± 5.5 nm for CSM, CSM@rtPA, and CSM@rtPA/L respectively, Fig. 1C, Additional file 1: Fig. S2 and Tables S2) also corroborates that colloidal stability is preserved along the synthetic and storage process. The morphology and size of CMS and rtPA loaded CSM (CSM@rtPA) formulations were also confirmed by scanning electron microscopy (STEM-in SEM) after negative staining with uranyl acetate (Fig. 1D; Additional file 1: Fig. S3). The diameter in size of CSM and CSM@rtPA samples obtained by STEM-in SEM micrographs (150–160 nm) agrees with the data obtained by NTA and DLS analysis. These results suggest that the rtPA encapsulation and the lyophilization process do not interfere with the nanocarrier morphology, size, and colloidal stability.

NTA data also reported the concentration of CSM and CSM@rtPA; and rtPA cargo loading (~ 0.1 mg rtPA/ mL, corresponding to ~ 20% of total rtPA added, Fig. 1E and Additional file 1: Fig. S4) was calculated by fluorescence measurements (Additional file 1: Fig. S4). Determining numbers of CSM per mL and rtPA concentration are critical parameters for the therapeutic dose of rtPA, CSM and CSM@rtPA administered in the in vivo experiments. The capacity of the rtPA loading on the lyophilized CSM@rtPA/L samples remains unaltered after the process (Fig. 1E and Additional file 1: Fig. S4).

Flow cytometry was next used to evaluate the biological features and platelet-mimicking functionalities of the CSM surface. The presence of specific cell membranes components such as phospholipids and specific surface markers was analyzed by specific fluorescence reporters and antibodies. First, flow cytometry analysis of CSM and CSM@rtPA dispersions revealed that all CSM nanocarriers were loaded with FITC-labeled rtPA, and all CSM@rtPA population is positive for the lipid staining by the CellMask reporter (Fig. 1F and Additional file 1: Fig. S5).

Translocation of specific platelet membrane surface markers, including transmembrane proteins such as CD42 (GPIbα) and CD47, was studied by immunostaining. Antibodies that recognize the extracellular domain of CD42 and CD47 were incubated with platelets and CSM@rtPA samples before and after lyophilization (CSM@rtPA/L). Platelets presented high expression of these two key surface markers which mediate collagen binding and increase immune compatibility. Flow cytometry data also showed expression of these markers on the CSM samples (Fig. 1G and Additional file 1: Fig. S6), suggesting that these transmembrane proteins were successfully transferred to the CSM surface and that the nanocarriers self-assembled with a right-side-out membrane orientation. In addition, the level of expression of CD42 and CD47 surface markers remains unaltered after the lyophilization process.

### Activity of encapsulated rtPA and in vitro studies

**Fig. 2:**
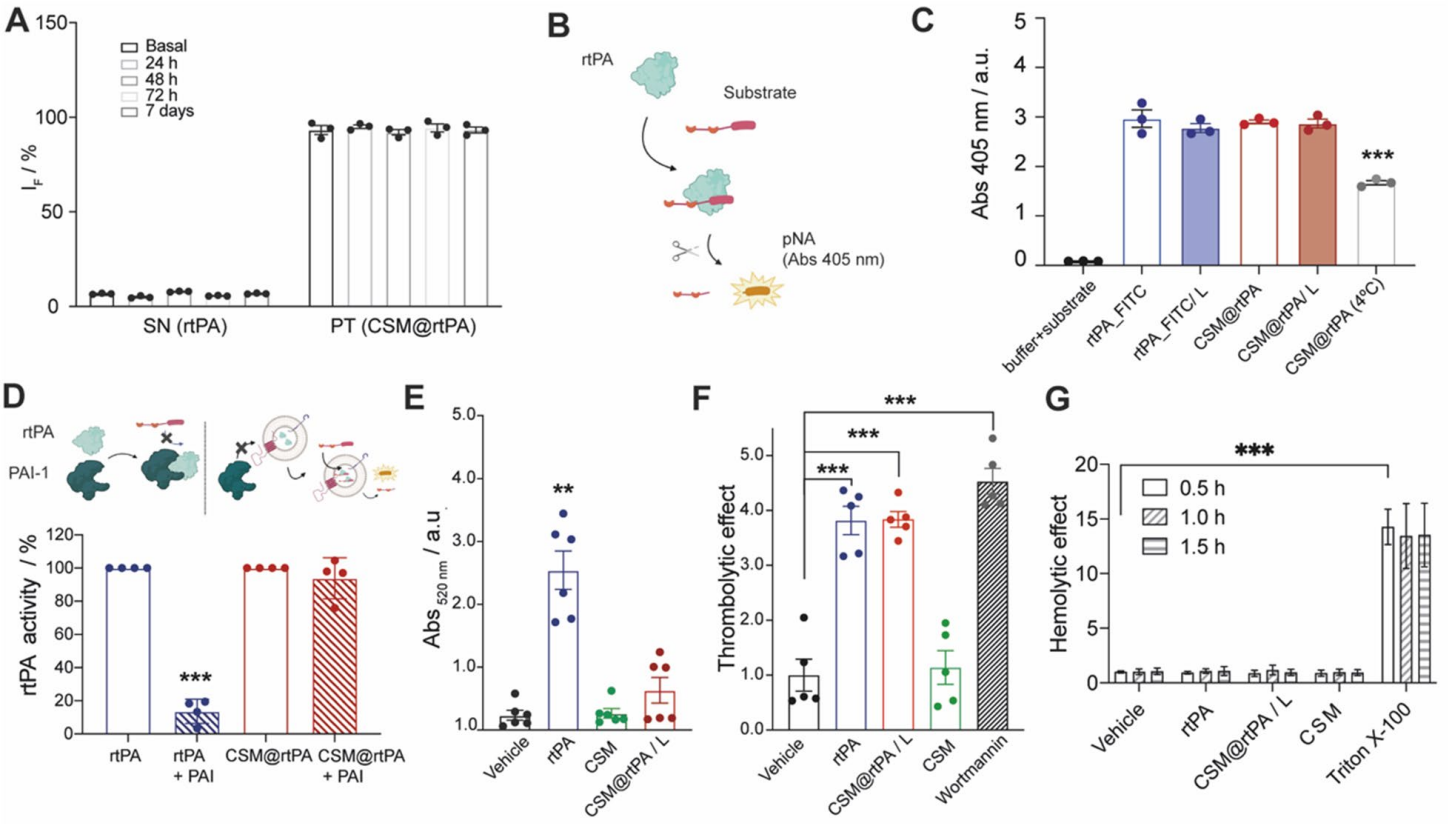
**A** Leaking assay performed at different time points: basal, 24 h, 48 h, 72 h, and 7 days after the synthesis. Data represent mean ± SEM (n = 3, independent samples). One-way ANOVA followed by post-hoc Dunnett’s multiple comparison test was used to perform statistical analysis compared to the IF of the corresponding control (basal). **B** Schematic representation of the rtPA amidolytic activity using a chromogenic substrate, which after hydrolysis by rtPA, the group pNA from the substrate is released. **C** The proteolytic activity of free rtPA and encapsulated rtPA (CSM@rtPA after storage by lyophilization process or storage at 4 ºC) in presence of the chromogenic substrate is recorded by measuring the absorbance at 405 nm over time. Data represent mean ± SEM (n = 3, independent samples). One-way ANOVA followed by post-hoc Dunnett’s multiple comparison test was used to perform statistical analysis compared with rtPA group (***P < 0.001). **D** The amidolytic activity is blocked when the rtPA is recognized by PAI-1. The proteolytic activity of free rtPA and encapsulated rtPA (CSM@rtPA) is determined in presence and absence of the PAI-1. Data represent mean ± SEM (n = 3, independent samples). One-way ANOVA followed by post-hoc Dunnett’s multiple comparison test was used to perform statistical analysis compared with the rtPA group (***P < 0.001). **E** Clots treated with the vehicle, rtPA (1 mg/kg), CSMs (number equivalent to the CSM@rtPA) and CSM@rtPA/L (equivalent to 1 mg/kg rtPA). Data represent mean ± SEM (n = 6, independent measurements per group of treatment). Statistical analysis was assessed by Kruskal–Wallis test followed by Dunn’s multiple test compared with the vehicle treatment group (**P < 0.01). **F** Thrombolytic effect displayed as fold change of clot mass loss normalized to average clot mass loss with PBS as control treatment. Treatments of either vehicle (PBS), rtPA, CSM and CSM@rtPA/L, or Wortmannin were injected via a microdialysis pump at a speed of 0.3 mL/min. Following treatment, vials were weighed again to determine final clot mass and total clot mass loss for each treatment. Data represents mean fold change ± SEM, (n = 5, independent measurements per group of treatment). One-way ANOVA followed by post-hoc Dunnett’s multiple comparison test was used to perform statistical analysis compared with the vehicle group (***P < 0.001). **G** Hemolytic effects of CSM and CSM@rtPA/L compared to free rtPA. Hemolytic effect displayed as absorbance fold change from mean PBS (vehicle) treatment at 0.5 h. RBC pellets treated with either vehicle, rtPA, CSM@rtPA/L, CSM, or 0.1% Triton X-100. Three biological replicates performed with three biological replicates each. Data represents mean absorbance fold change ± SEM, (n = 6, independent measurements per group of treatment). One-way ANOVA followed by post-hoc Dunnett’s multiple comparison test was used to perform statistical analysis compared with the vehicle group (***P < 0.001)

The encapsulation stability of FITC labeled rtPA was studied by fluorescence quantification of the rtPA leaking over time for 1 week. After CSM@rtPA synthesis, we took samples at different time points: basal, 24 h, 48 h, 72 h and 7 days. We centrifuged the samples and measured the fluorescence intensity (IF) in the precipitate (containing CSM@rtPA) and supernatant. Leaking assay demonstrated the stability of the rtPA encapsulation into CSMs for 7 days (Fig. 2A).

To evaluate the amidolytic activity of the encapsulated rtPA we employed a chromogenic peptide substrate that is permeable to the nanocarrier surface and selectively hydrolyzed by rtPA, releasing the chromophore pNA (that absorbs at 405 nm) (Fig. 2B). As shown in Fig. 2C (and Additional file 1: Fig. S7), the enzymatic activity of rtPA encapsulated in freshly prepared CSM@rtPA and lyophilized CSM@rtPA/L samples remained similar to rtPA control levels. However, if the CSM@rtPA samples are storage at 4 ºC the enzymatic activity of rtPA significantly decreases till half after 2–3 days (Fig. 2C; Additional file 1: Fig. S7). Therefore, rtPA encapsulated in the CSM did not lose its amidolytic activity when is storage after the lyophilization process. Subsequently, all in vitro experiments reported in Fig. 2 were performed with lyophilized CSM@rtPA samples.

The natural inhibitor of rtPA, plasminogen activator inhibitor 1 (PAI-1), was incubated with the samples, and the enzymatic activity of rtPA was analyzed. PAI-1 is not permeable to the nanocarrier surface. Notably the amidolytic activity of free rtPA control completely decreased in presence of the PAI while the encapsulated CSM@rtPA remained active (Fig. 2D).

The study of the fibrinolytic activity of the free and encapsulated rtPA was carried out in rat clots. Clots were artificially pre-formed and treated with vehicle, rtPA (100 µg/mL), CSM@rtPA (100 µg/mL), and CSMs (same number of particles as in CSM@rtPA). rtPA treatment increases the digestion of the clots after 30 min. However, CSMs and CSM@rtPA did not affect the fibrinolytic activity along this time, demonstrating that rtPA inside the CSM@rtPA was protected for shorter times (Fig. 2E).

We next wanted to determine if encapsulated rtPA (CSM@rtPA) is released over time and retained its thrombolytic activity. Whole blood from rat pups was incubated in vials for 45 min at 37 °C to form clots. The vials containing clots were attached to a microdialysis which injected treatments solutions at a rate of 300 µL/min, mimicking cerebrospinal (CFS) flow rate. Treatments used were PBS (as a negative control, vehicle), rtPA control, CSM@rtPA, CSM, and wortmannin (a PI3K-Akt pathway inhibitor [27] as a positive control of clot degradation). We found that after 1 h of treatment, CSM@rtPA significantly increased clot lysis compared to control, similar to rtPA control and Wortmannin do (increased clot lysis by 3.82 ± 0.58-fold, 3.84 ± 0.32-fold, and 4.53 ± 0.53-fold) (Fig. 2F). rtPA did not lose its thrombolytic effect in vitro when encapsulated in the CSM, suggesting a promising biomimetic platform for rtPA cargo delivery to the clot site.

As rtPA is administered intravenously, we wanted to investigate if either empty or loaded CSMs had a deleterious effect on a key component in blood, red blood cells (RBCs). Hemolysis was assessed by measuring the absorbance at 405 nm of the supernatant of RBC pellets following treatment at either 0.5, 1.0, and 1.5 h with either vehicle, rtPA, CSM, CSM@rtPA, or 0.1% Triton X-100 (a common detergent used to lyse cells and permeabilizing cell membranes used as a positive control). The results demonstrated no significant changes in the level of hemolysis after treatment with rtPA, CSM or CSM@rtPA compared to the vehicle (Fig. 2G). In contrast, Triton X-100 significantly hemolyzed RBCs at all three time points. While rtPA was not expected to cause significant hemolytic effects as it is endogenously produced by endothelial cells and released into the bloodstream, it was important to assess whether the carrier itself exerted a hemolytic effect.

Taken together, these results indicate that the rtPA have been successfully encapsulated in the CSM@rtPA samples and storage without impairing its biological function.

Before evaluating the in vivo activity of the nanocarriers, we performed in vitro analysis of CSM and CSM@rtPA cytotoxicity in human macrophage (differentiated THP-1 cells) and human microglia (HMC3 cells). Human HMC3 cells and THP-1 differentiated macrophages were treated for 24 h with increasing doses of rtPA, CSM@rtPA, and CSMs. Results showed that neither the empty CSM nor rtPA loaded CSMs produced significant decreases in cell counts in either microglia cells or macrophages (Fig. S8). This was a necessary step to ensure that the nanocarriers did not have a detrimental effect on the cell viability of two of the main cell players in stroke within the clinically relevant intravenous rtPA concentrations used in stroke patients.

### Safety analysis CSM@rtPA in healthy animals

**Fig. 3:**
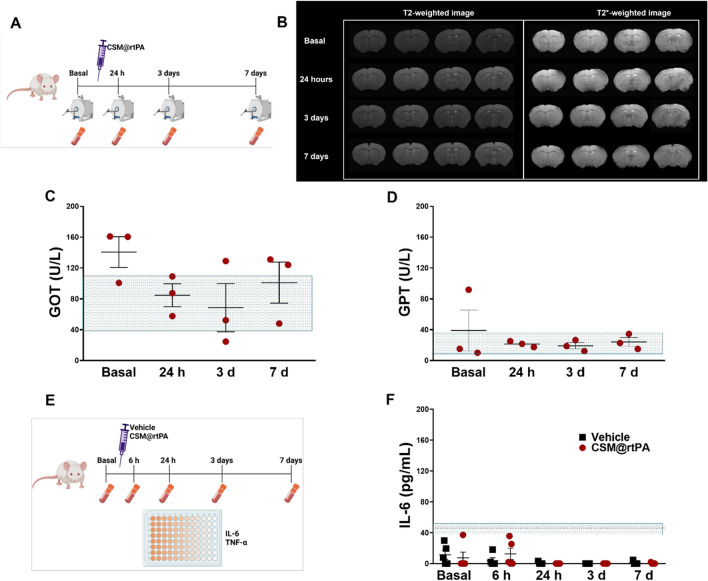
**A** Schematic representation of the CSMs safety. Blood samples and MRI were analyzed before and 24 h, 3 days and 7 days after the CSM@rtPA administration. **B** T2 and T2*-weighted images MRI showed that hemorrhages or ischemic damages were not found. Furthermore, the analysis of **C** GOT and **D** GPT as hepatic markers demonstrated that CSM@rtPA did not provoke toxicity in the liver and kidneys. These data are represented as mean ± SEM (n = 3 per group of treatment). Statistical analysis was assessed by Kruskal–Wallis test followed by Dunn’s test compared with the basal. **E** To study the inflammatory response alteration blood samples were extracted before and 6 h, 24 h, 3 days, and 7 days after the CSM@rtPA administration. Levels of IL-6 (**F**) did not change over the days. The dotted lines represented the detection range of the ELISA kit. These data are represented as mean ± SEM (n = 5 per group of treatment). Statistical analysis was assessed by Kruskal–Wallis test followed by Dunn’s multiple test compared with the corresponding basal timepoint

In order to evaluate the biocompatibility and to study whether the CSM@rtPA provokes any cerebral damage, hepatic or renal failure, and inflammatory response, brain MRI scans and blood analysis were performed in healthy animals. The analysis of the MRI, hepatic (GOT, GPT) and renal (creatinine) markers were performed before and 24 h, 3 days, and 7 days after the treatment administration (Fig. 3A). MRI analysis demonstrated that CSM@rtPA did not provoke any brain visible lesion (Fig. 3B). Any signs of ischemic or hemorrhagic damage should appear as hyper-signals (white spots) or hypo-signals (black regions) respectively in the brain. Levels of GOT and GPT, remained within normal parameters following treatment (Fig. 3C, D). Creatine levels were below the detection limit in our analytical system, and therefore, below the levels of renal toxicity.

The inflammatory response was analyzed before and 6 h, 1 day, 3 days and 7 days after the administration of vehicle and CSM@rtPA in healthy animals (Fig. 3E). The profile of IL-6 levels was similar in both groups (Fig. 3F). In the case of the TNF-α levels, all measurements were below the detection range of the ELISA (9.1 pg/mL).

### In vivo pharmacokinetic analysis and bleeding time evaluation of CSM@rtPA

**Fig. 4:**
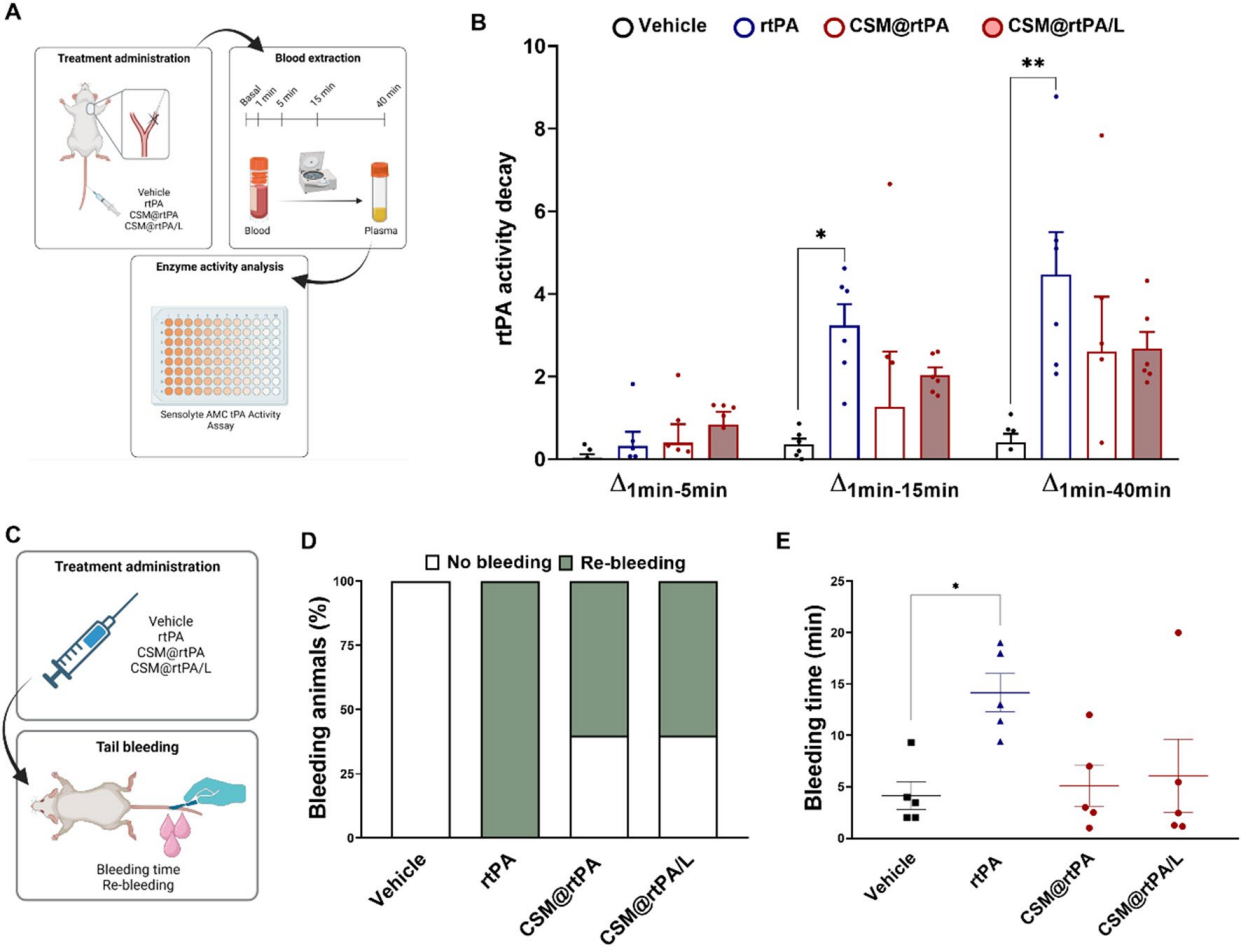
**A** Schematic representation of the pharmacokinetic study. To measure rtPA plasmatic activity, blood samples were extracted before (basal) and 1, 5, 15 and 40 min after the treatment administration. **B** Variation of plasmatic tPA activity during a period of 40 min after treatment administration of groups treated with vehicle, rtPA (1 mg/kg as bolus), fresh and lyophilized CSM@rtPA (1 mg/kg as bolus). Data are represented as mean ± SEM (n = 6 per group of treatment). Statistical analysis was assessed by Kruskal–Wallis test followed by Dunn’s multiple test comparing all the groups at the same timepoint (*P < 0.05; **P < 0.01). **C** Schematic representation of the bleeding assay. After the administration of the treatments (vehicle, 1 mg/kg rtPA, 1 mg/kg CSM@rtPA and 1 mg/kg CSM@rtPA/L) the tail was cut off. **D** Percentage of the animals in which re-bleeding occurred and **E** the bleeding time was quantified. Data represent mean ± SEM (n = 5 per group of treatment). Statistical analysis was assessed by a one-way ANOVA followed by Tukey’s multiple comparison test comparing all the groups (*P < 0.05)

Pharmacokinetic rtPA plasmatic activity after administration of CSM@rtPA was studied by extracting blood samples before and 1, 5, 15, and 40 min after the administration in healthy animals (Fig. 4A). The following experimental groups were included in the analysis: (1) vehicle (saline); (2) 1 mg/kg of rtPA as bolus (used as control free-rtPA group); (3) CSM@rtPA (corresponding to 1 mg/kg of rtPA) as bolus; (4) CSM@rtPA/L (corresponding to 1 mg/kg of rtPA) as bolus. While vehicle did not affect the basal plasma fibrinolytic system, other three treatments with free or encapsulated rtPA (1 mg/kg) caused an immediate increase in the plasma tPA activity. Analysis of rtPA decay activity calculated as the difference between rtPA activity at 1 min and 5, 15, 40 min after treatment administration showed that the decay activity of rtPA is faster in the group treated with free rtPA compared to both groups of CSM@rtPA samples (fresh and lyophilized) (Fig. 4B). This evidence the efficient encapsulation of rtPA, the protection of the enzyme activity against blood inhibitors and the sustained release of the encapsulated drug. Tail bleeding assay, used as common method to evaluate the pharmacological effects of hemostatic agents, was performed in an independent group of animals to examine the re-bleeding rate and the time of bleeding after administration of different treatments: (1) vehicle (saline); (2) 1 mg/kg of rtPA as bolus; (3) CSM@rtPA (corresponding to 1 mg/kg of rtPA) as bolus; (4) CSM@rtPA/L (corresponding to 1 mg/kg of rtPA) (Fig. 4C). According with other studies, is known that administration of free rtPA reduces stability and clot formation, which is reflected in re-bleedings events in all animals treated, while only 40% of animals in the group treated with CSM@rtPA and CSM@rtPA/L presented re-bleeding (Fig. 4D). When we evaluated the bleeding time in these animals, it was observed that times were increased in the group treated with rtPA compared with the vehicle group (14.2 ± 1.9 min vs 4.5 ± 1.7 min; P < 0.05). Interestingly, when the animals were treated with CSM@rtPA and CSM@rtPA/L, the bleeding time decreased to 5.10 ± 2.0 min and 6.1 ± 3.6 min, respectively (Fig. 4E). These last data are in line with Fig. 4B, which indicates the sustained release of the encapsulated drug, reducing the bleeding time and, potentially, the risk of bleeding.

### Therapeutic effect of CSM@rtPA in stroke animal models.

**Fig. 5:**
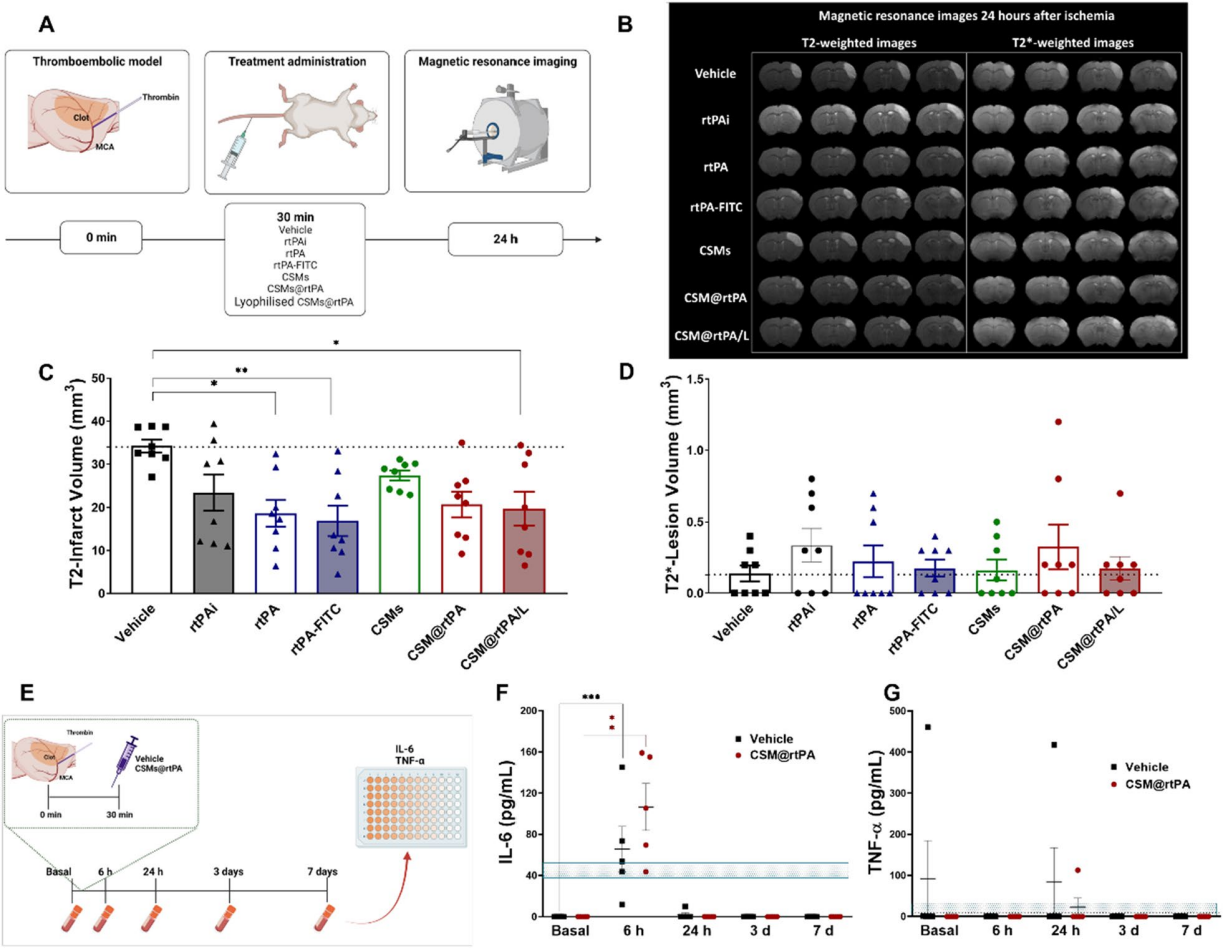
**A** Schematic representation of the experimental in vivo study. Animal ischemic model was induced by the local injection of thrombin in the middle cerebral artery (MCA). Treatments were injected through the vein 30 min after ischemia induction. Vehicle was used as control group. rtPAi (1 mg/kg as bolus and infusion), rtPA (1 mg/kg), rtPA-FITC (1 mg/kg), CSMs, CSM@rtPA (1 mg/kg as bolus) and CSM@rtPA/L (1 mg/kg as bolus) was administered as bolus. Infarct volumes and hemorrhages were evaluated at 24 h after ischemia by magnetic resonance imaging (MRI). **B** Ischemic lesion (white brain region) determined by T2-weighted at 24 h after the treatment administration in the experimental groups. Furthermore, the presence of hemorrhages was studied using the T2*-weighted images. **C** Analysis of the infarct volume at 24 h after the treatment administration. Dotted lines represent the median of the vehicle group. Dataare represented as mean ± SEM (n = 8 per treatment group). Statistical analysis was assessed by the on-way ANOVA followed by post-hoc Tukey’s test comparing all the groups against each other (*P < 0.05; **P < 0.01). **D** Analysis of hemorrhagic transformations evaluated by MRI T2*-weighted images. Dotted lines represent the median of the vehicle group. Dataare represented as mean ± SEM (n = 8 per treatment group). Statistical analysis was assessed by Kruskal–Wallis test followed by Dunn’s test comparing all the groups against each other. **E** Schematic representation of the inflammatory response study in ischemic mice. Treatments (vehicle and CSM@rtPA (1 mg/kg)) were administered 30 min after ischemia. Blood samples were extracted before and 6 h, 24 h, 3 and 7 days after the treatments to analyze **F** IL-6 and **G** TNF-α. Dotted lines represented the normal values of both inflammatory markers. These data represent mean ± SEM (n = 5 per group of treatment). Statistical analysis was assessed by Kruskal–Wallis test followed by Dunn’s multiple test compared with the corresponding basal timepoint (**P < 0.01; ***P < 0.001)

Therapeutic effect of CSM@rtPA and CSM@rtPA/L was finally evaluated in a thromboembolic model that is a widely accepted tool to evaluate the recanalization effects of rtPA [28]. In this model, thirty minutes after thrombin injection, different treatments were administered in the tail vein: (1) vehicle (saline); (2) 1 mg/kg of rtPA administered 10% as bolus and 90% as infusion (rtPAi), as used in the clinical practice; (3) 1 mg/kg of rtPA as bolus; (4) 1 mg/kg of rtPA-FITC as bolus (5) CSMs without rtPA (to the same number of CSM@rtPA) as bolus; (6) CSM@rtPA (corresponding to 1 mg/kg of rtPA) as bolus; (7) CSM@rtPA/L (corresponding to 1 mg/kg of rtPA) as bolus (Fig. 5A). Of note, experimental group (4) was included in the analysis to confirm that FITC did not interfere with the thrombolytic activity. FITC was used as a label to estimate the encapsulation of rtPA and use a dose of 1 mg/kg in the respective treated groups.

To measure the infarct volume and assess the presence of hemorrhages, MRI was performed 24 h after the surgery (Fig. 5B). Figure 5C shows that rtPA, rtPAi and rtPA-FITC treatments reduced the infarct volume compared to the vehicle group (rtPA group: 18.6 ± 3.1 mm^3^; rtPAi group: 23.4 ± 4.2 mm^3^; rtPA-FITC group: 16.9 ± 3.6 mm^3^; vehicle group: 34.2 ± 1.5 mm^3^). When CSMs without rtPA were administered the infarct volume did not decrease the lesion (27.4 ± 1.2 mm^3^). CSM@rtPA and CSM@rtPA/L reduced infarct volume significantly, compared to the vehicle group, achieving 20.7 ± 3.0 mm^3^ (P < 0.05) and 19.7 ± 4.0 mm^3^ (P < 0.01), respectively. In general, similar response on infarct reduction was observed between rtPA solution (infusion and bolus) and bolus injection of CSM@rtPA samples (fresh and lyophilized). In parallel, T2*-weighted images were used to evaluate the HT, observed as hyposignals due to the blood leakage and iron accumulation in the brain tissue [29, 30]. Differences between groups were not evidenced in terms of risk of HT (Fig. 5D).

In an independent experimental group, IL-6 and TNF-α, used as markers of inflammation after stroke, were analyzed in ischemic mice before and after the treatment with the vehicle and CSM@rtPA (Fig. 5E). Data shown in the Fig. 5F, G indicate that no relevant inflammatory events were associated with the treatments used.

## Discussion

The use of nanotechnology has been widely explored in the field of stroke with promising advances in the development of new platforms for the improvement of drug efficacy, safety, and prolonging the effect of thrombolytic therapeutics, such as rtPA [20]. However, despite the positive results reported thus far, none of these approaches have translated to clinical practice.

In previous studies, we have already reported the design of novel sonosensitive polymer-based capsules synthesized by the layer-by-layer technique for rtPA encapsulation [31, 32]. These capsules also incorporated iron oxide nanoparticles to make them suitable for magnetic resonance imaging (MRI) and gelatin as an outer layer in the nanocarrier´s surface to target the clot to the ischemic region. We demonstrated that this system protects rtPA from its main blood inhibitor (PAI-1) and responds to ultrasounds for controlled drug release. However, despite its promising efficacy and safety in healthy animals, when they were administered in stroke animal models for therapeutic analysis, these capsules appeared aggregated in the ischemic brain region, increasing the lesion, and limiting the thrombolytic effect of rtPA. Large particle size (~ 600 nm) and poor elasticity in combination with the ischemic damage in the structural integrity of brain vasculature could explain this particle accumulation. This previous experience led us to postulate that the use of cell-derived nanocarriers of smaller size and increased elasticity, could be more appropriate for pathologies with vascular impairment as against stroke. To evaluate this hypothesis, we have developed thrombolytic nanoparticles derived from platelets, defined as CSM@rtPA, with a size of 200 nm. To simplify the synthesis and improve the elasticity of particles, rtPA was directly encapsulated in cellsomes derived from platelets, without the use of encapsulation in synthetic particles, as other studies have reported before [33].

One of the major goals in this synthesis process was to confirm the preservation of platelet membrane and orientation of cell surface proteins in the outside region of the particles. By flow cytometry, we could confirm that surfaces markers of platelets involved in thrombus targeting and immune compatibility, such as immunomodulatory protein, CD47, and transmembrane glycoproteins, GPIba (CD42), were retained on the CSM@rtPA nanocarrier surface.

A majority of commercially available nanomedicine formulations are in liquid suspensions. Storage at low-temperature could promote particle thermodynamic stability loss and lead to aggregation over time. A freeze-drying process, which involves water removal, helps to minimize potential particle aggregation and drug degradation. We have demonstrated that lyophilization process, employed to guarantee long-term storage conditions of CSM@rtPA, did not interfere with their physico-chemical and biological properties. Drug activity was preserved even after sample reconstitution. This process is robust and highly reproducible; no significant differences between batches were observed. This significant improvement enabled the synthesis of large quantities of CSM@rtPA and the use of the same batches of nanoformulations during the project and in different laboratories. More importantly, the success of lyophilization simplifies the process of scaling-up and the use of these particles in future clinical studies.

Complementary characterization analysis confirmed that CSM provides protection and long stability of thrombolytic drug against the natural blood tPA inhibitor, PAI-1. The in vitro clot assays also demonstrated that the thrombolytic ability of rtPA on clots is retained in the CSM@rtPA lyophilized samples without incurring increased hemolytic activity on RBCs. Some previous studies designed with the same purpose have opted to incorporate the thrombolytic drugs in the membrane surface, an approach that allows for direct interaction between the thrombolytic drug and the clot, allowing for faster degradation arterial recanalization, however this approach can led to rapid inactivation by PAI-1 and significantly reduces the half-life of the enzyme in blood after administration [20].

In vivo experiments developed first in healthy animals, showed that the designed CSM were biocompatible and safe as brain lesions were not detected, no hepatic or renal damage were observed, and inflammatory response was not provoked. Furthermore, in line with the in vitro analysis, CSM nanocarriers were capable of prolonging rtPA retention and stability by avoiding degradation from PAI-1 and reducing the systemic bleeding. No differences were also observed between “fresh” and reconstituted lyophilized particles. This finding reaffirms that the drug's efficacy remains intact when it is encapsulated within platelet-derived nanocarriers, even in the form of a dried powder.

Comparative therapeutic efficacy between CSM@rtPA and lyophilized particles were finally tested in a thromboembolic model of stroke in mice in which local injection of thrombin is used to create an in situ clot and occlusion of the middle cerebral artery; a model that is rtPA-sensitive and widely accepted to evaluate the recanalization effects of rtPA [28]. rtPA encapsulation in platelet derived CSMs showed to be as effective as free rtPA at the same concentration without increasing the risk of hemorrhagic transformations and provoking an inflammatory response.

Despite the similar response observed between rtPA (infusion and bolus) and CSM@rtPA bolus injection (fresh and lyophilized), these results are very promising from a clinical point of view. The free rtPA infusion protocol is only approved for clinical use, not for bolus administration due to the risk of bleeding. In this context, a similar effect between the bolus administration of rtPA particles and the perfusion of free rtPA represents an advantage in favor of the particles, as it simplifies administration. A single bolus is easier and less time-consuming in an emergency setting than a treatment perfusion, particularly in stroke patients. In addition, the avoidance of continuous infusions may also reduce the need for medical supervision during treatment administration and improve door-to-needle time.

In addition, our results demonstrate the biocompatibility of these CSMs in a complex and vascular pathology as stroke. Stroke causes vessel wall impairment and a reduction of the microvasculature lumen in the ischemic region that favors particle aggregation and increases the risk of secondary ischemic lesions, as we have previously reported when submicrometric capsules loaded of rtPA was used [32]. The use of CSMs for rtPA encapsulation clearly overcome the aggregation events in the ischemic regions and allow a controlled and sustained release of the thrombolytic drug with effective recanalization.

## Conclusions

There are very few studies like this reporting the characterization, loading capacity, and efficacy of lyophilized thrombolytic particles for stroke treatment. Optimization of formulations and the lyophilization cycle is crucial to guarantee the preservation of nanoparticles and translate the research to the clinical application. Our findings show that lyophilization of the cellsomes with rtPA allowed their prolonged stability, did not cause hemorrhage risk and did not decrease rtPA biological activity meanwhile maintain its in vivo effectiveness. Finally, this study shows an effective way of platelet biomimetic nanomedicine application for precise thrombolytic treatment of acute stroke suggesting the use of similar nanostructures for stroke therapy particularly as a component of a combination therapy.

## Methods

### Synthesis of CSM@rtPA

The synthesis of 200 nm sized nanovesicles called cellsomes (CSMs) derived from membrane fragments of platelets was carried out following the protocol described in the literature [26]. Human Platelet Concentrate (#SER-PCEX, 2 batches FDA registration numbers 3007651216 and 1072425) were purchased from Zen-bio^©^, aliquoted (5 mL about 10^9^–0^11^ cells each) and stored at 4 ºC until use. One aliquot was centrifuged at 500*g* for 5 min, resuspended in phosphate-buffered saline (PBS, pH 7.4 Thermo Fisher #14190169) and centrifuged at 600*g* for 5 min. The cell pellet was resuspended in 10 mL of hypotonic buffer (0.25 × PBS) containing 1 × protease inhibitor cocktail (PIC, Sigma-Aldrich™ #P2714-BTL) and left at 4 ºC for 10 min. The cell lysis was carried out using a freeze–thaw method consisting of 4 cycles of freezing in liquid nitrogen for 1 min followed by thawing at 37 °C for 10 min. The solution was subjected to ultrasounds in an ultrasonic bath for 10 min. The solution was then subjected to several centrifugation steps to purify the cell membrane fragments. First the solution was centrifuged at 700*g* for 10 min at 4 °C to discard the nuclei or whole cells. The obtained supernatant was precipitated by centrifugation at 15,000*g* for 30 min at 4 °C. Finally, the pellet containing cell membrane fragments was resuspended in 1 × PBS and a mechanical extrusion process using an Avanti® Mini extruder with 800 nm polycarbonate membrane was applied to favor the self-assemblage of the membrane fragments obtained into CSMs. The sample was subjected to ten consecutive cycles of extrusion.

For cargo loading on CSM, 5 mg of commercially available recombinant tissue plasminogen activator (rtPA) (Actilyse^®^) were labeled with fluorescein isothiocyanate isomer I (FITC) by mixing 25 FITC/rtPA (mol/mol) in 1 × PBS + l-Arginine (3.5 mg/mL) [31]. The reaction was left overnight at 4 ºC and then purified from the excess of FITC by size exclusion chromatography using a PD-10 desalting column. Then, the fluorescently labeled rtPA was recovered in the second fraction collected from the PD-10 column. Bradford assay (Pierce TM Coomassie Plus Assay Kit; ThermoFisher #23236) was used to quantify FITC-labeled rtPA concentration. Once the protein concentration is determined, the rtPA is loaded in the CSMs. The sample of FITC labeled rtPA with a known concentration was used to perform a calibration curve of protein concentration *versus* fluorescence signal (λ_exc./em._ 485/535 nm). This calibration curve was used to determine the final rtPA-FITC loading onto the CSMs. Then, 0.4 mL of the purified rtPA-FITC (at 1 mg/mL) was added to 1 mL of CSM in PBS buffer. The solution was sonicated for 15 min, and another extrusion cycle was performed. Finally, the sample was purified from the excess of rtPA-FITC by centrifugation (1 h, 70,000 g, 4 ºC). CSM@rtPA was finally resuspended in 1 mL of 1 × PBS.

Lyophilization, also known as freeze-drying process, was used as a preservation process to long-term storage of CSM@rtPA samples. After preparation, samples dispersed in PBS buffer were subjected to lyophilization using a freeze dryer (Lyovapor™ L-300 Büchi) and stored at −20 ºC for months.

### Physico-chemical characterization of CSM@rtPA

Dynamic light scattering (DLS) was used to characterize the colloidal properties of the CSMs and determine the hydrodynamic size of the samples. The solutions were analyzed in 1 × PBS by using a DLS Malvern Zetasizer Nano ZSP (Malvern Instrument Ktd) equipped with a 10 mW He–Ne laser operating at a wavelength of 633 nm laser and fixed scattering angle at 173º. All measurements were carried out at 37 ºC. DLS distributions were obtained recording 3 measurements for each sample. Then the average hydrodynamic size distribution is calculated from 3 independent samples.

Nanoparticle tracking analysis (NTA) was used to characterize the colloidal properties of the CSMs and determine the CSMs concentration in solution. All the samples were analyzed in 1X PBS by using a NanoSight NS300 (Malvern Instrument Ktd) equipped with a 405 nm laser. All measurements were carried out at 25 ºC. CSMs were diluted 1:1000 in MilliQ water (200 nm filtered) to a final volume of 1 mL and loaded in the measurement chamber with a flow rate of 50 μL/min. Flow mode measurements were obtained recording 3 videos of 60 s for each measurement (with 10–100 particles/frame). Then the average size of CSM diameter is calculated from 3 independent samples.

Scanning transmission electron microscopy, STEM-in-SEM analysis allowed the characterization of the structural properties of CSMs. CSMs solution was stained by using a 2% uranyl acetate solution. The CSM sample (2 μL) was deposited from a dilute solution onto a 3–4 nm thick film of amorphous carbon supported on a 400-mesh copper grid (Ted Pella Inc., #01822-F). All images were obtained using a scanning transmission electron microscope ZEISS FESEM ULTRA Plus.

### Cargo loading quantification

The encapsulated FITC labeled rtPA into the CSM was quantified by fluorescence measurement using a Microplate reader (TECAN, Infinite^®^ 200 PRO) equipped with monochromator-based optics and wavelength selection between 280 and 850 nm (λ_exc./em._ 485/535 nm). The cargo concentration was determined by interpolation of the measured fluorescence intensity in the analytical calibration curve. The loading efficiency (% LE) was determined as the mass of rtPA in purified CSM@rtPA divided per mass of total rtPA added.

### Biological characterization of CSM@rtPA

Flow cytometry (FC) measurements were carried out to analyze the biological characteristic of the CSM. First the lipidic composition of the CSM samples were corroborated by staining the sample with 5 mL of CellMask™ DeepRed (1 ×), a specific dye that binds to lipid molecules. Then, fluorescently antibodies, human anti-CD47 APC conjugated Antibody (FAB4670A, R&D Systems) and human antiCD42b/GPlbα APC conjugated Antibody (FAB4067A, R&D systems) were incubated with platelets and with CSMs (10 µL of antibody stock solution /10^6^ cells) and the samples were analyzed by flow cytometer. A CytoFLEX flow cytometer (Beckman Coulter), using a blue laser emitting at 488 nm and a red laser emitting at 642 nm was used. Background measurements in PBS buffer were carried out before each measurement. Forward (FSC) and side scattering (SSC) signals were recorded to gather information of the platelets and CSM dispersions. CSM samples were acquired using a low flow rate and 100,000 events were recorded for each measurement. Platelet samples were acquired using a medium flow rate and 50,000 events were recorded for each measurement. The fluorescence signal was measured from the corresponding channel: Green channel (blue laser excitation) (525/40 nm) for FITC-rtPA signal and Red channel (red laser excitation) (660/10 nm) for Cell Mask DeepRed (λ_ex/em_ 660/675 nm) and APC labeled antibodies (λ_ex/em_ 650/660 nm) signals.

### rtPA leaking assay

The encapsulation stability was studied with an indirect measurement of the mass cargo based on fluorescence. The experiment was performed at 37 °C as follows. After CSM@rtPA synthesis, we take samples in plasma at different time points: basal, 24 h, 48 h, 72 h and 7 days. We centrifuged the samples and the fluorescence intensity (I_F_) in the precipitate (containing CSM@rtPA) and supernatant was measured.

### rtPA activity

Tissue plasminogen activator chromogenic substrate (Sigma-Aldrich™ #T2943, CH_3_SO_2_-D-HHT-Gly-Arg-pNA AcOH) was used to determine rtPA activity once encapsulated in CSMs. The proteolytic activity of rtPA is determined by the hydrolysis of the chromogenic substrate and the release of the free pNA (p-Nitroaniline absorbance at 405 nm). 10 µL of 20 µg/mL free rtPA or rtPA loaded into CSMs were diluted in 200 µL of 1 × PBS + l-Arg (3.5 mg/mL) and 48 µL of the chromogenic substrate was added subsequently. The kinetic measurement of absorbance was then determined with a plate reader (TECAN, Infinite^®^ 200 PRO) at 405 nm for 12 h (kinetic time point = 1 h). Since CSMs should prevent rtPA endogenous inactivation, rtPA activity was also tested in the presence of plasminogen activator inhibitor (PAI) (2 µL of PAI solution 0.25 mg/mL).

### Clot disaggregation assay

The study of the fibrinolytic activity of free and encapsulated rtPA was carried out in rat clots. Clots were artificially pre-formed in 96-well plates after drawing blood from rats; we added 75 µL of freshly extracted blood to each well. After 1 h to allow clotting, we added the different samples: vehicle, rtPA (100 µg/mL), CSM@rtPA (100 µg/mL), and CSMs. In the groups treated with the CSMs we added the same number as CSM@rtPA. After 30 min of treatment, the supernatant (SN) was extracted and the absorbance, at 540 nm, was measured.

### Thrombolytic assay

A CMA100 Microdialysis pump was used to mimic the speed of CSF in the brain (around 0.3 mL/min) due to its variable injection speed. 100 µL of whole blood was used from sacrificed 2-day old rat pups and connected to a pre-weighed vial connected to the microdialysis pump. The blood was incubated at 37 °C for 45 min to allow for the formation of clots. After which, the non-clotted fluid was aspirated from the vials, and weighed two of each calculate the initial clot weight. Pre-incubated treatments were added via syringes attached to the microdialysis pump and injected at a speed of 0.3 mL/min for an hour. Treatments used were PBS (as vehicle), 0.2 µg/mL rtPA, CSM@rtPA, CSM, or Wortmannin (as a positive control of clot degradation). Vials containing the formed clots were aspirated and weighed to determine the difference in clot mass before and after treatment. Four biological replicates were performed.

### Hemolytic assay

100 µL of whole blood from 6-month-old male mice was placed in 1.5 mL vials along with 500 µL PBS and centrifuged at 500*g* for 5 min to form a pellet of red blood cells. Following centrifugation, cells were washed with 0.5 mL PBS twice. 1 mL of treatment was added to different vials and incubated for 1 h, after which, vials were centrifuged again at 500*g* for 5 min. 50 µL of supernatant was collected from each vial and the hemoglobin (released from destroyed RBCs) was assessed using a microplate reader at absorbance 405 nm (BioChrom EZ Read 2000).

### Cell cultures

Human microglia clone 3 (HMC3) cells were obtained from the American Tissue Culture Collection (ATCC). Cells were maintained in Dulbecco’s Modified Eagle Medium (DMEM; Gibco, #11995-065), supplemented with 5% fetal bovine serum (FBS; Wisent #080-450) and 1% Penicillin–Streptomycin (P-S; Invitrogen, #15140-122). THP-1 human monocyte cells were obtained from ATCC. THP-1 cells were cultured in Roswell Park Memorial Institute 1640 (RPMI 1640; Gibco, #11835-030) containing 10% (FBS), 1% penicillin–streptomycin (P/S), and 50 µM of β-mercaptoethanol (Bio-Rad, #161-0710) in a 75 cm^2^ vented cell culture flask (Sarstedt, 83.3911.002). Differentiation of THP-1 monocytes into macrophages was induced over a period of 5 days with phorbal-12-myristate-13-acetate (PMA; Abcam, ab120297) at a concentration of 75 ng/mL. Differentiating THP-1 cells were refreshed with media containing PMA on the third day. HMC3 cells were kept at passages below 35, while THP-1 passages were limited to 12. Both cell types were incubated at 37 °C, 5% CO_2_, and atmospheric O_2_.

### Cell counting

Cell nuclei of HMC3 or THP-1 cells were labelled with 10 µM Hoechst 33342 for 30 min at 37 °C following treatment and imaged with a fluorescent microscope (Leica DMI4000 B). Cells were counted using the Cell Counter plugin in FIJI (ImageJ).

### Animal procedures

Male Swiss mice (Harlan Laboratories) weighing 25–30 g were used for in vivo assays. Mice were kept in separate rooms both under controlled conditions of temperature (22 °C ± 1 °C) and humidity (60% ± 5%) with a 12/12-h light/dark cycle for a week prior to surgery and up to 7 days after surgery. They had access to food and water ad libitum*.*

All the procedures were performed under anesthesia induced by the inhalation of 5% sevoflurane in a nitrous oxide/oxygen mixture (70/30). Rectal temperature was monitored and maintained at 37 °C ± 0.5 °C by using a feedback-controlled heating system. At the end of the procedures, mice were sacrificed under deep anesthesia (8% sevoflurane). was approved by the Animal Experimental Committee of the University of Santiago de Compostela, Spain. The animal experiments were conducted under the procedure number: 15011/2022/005 according to the Spanish and European Union rules (86/609/CEE, 2003/65/CE, 2010/63/EU, RD 1201/2005 and RD 53/2013).

### CSM safety in vivo

In order to study whether the CSM@rtPA provokes any damage in the brain, hepatic or renal failure or inflammatory response, blood extractions and MRI were performed.

In one hand, blood samples and MRI were carried out before and 24 h, 3 and 7 days after the administration. In the case of MRI study, both T2 and T2*-weighted sequences were analyzed to search ischemic or hemorrhagic transformation, respectively. Furthermore, three standard toxicity markers were evaluated: GOT and GPT as hepatic markers, and creatinine as renal marker of toxicity. The analysis was conducted with Reflotron® (Roche) by adding 32 µL of blood to reactive strips for GOT, GPT and creatinine.

In other hand, the inflammatory response was studied measuring the interleukin-6 (IL-6) and tumor necrosis factor (TNF-α) levels before and 6 h, 24 h, 3 and 7 days after the CSM@rtPA administration. The measurement of IL-6 (ab222503, Abcam) and TNF-α (ab208348, Abcam) was carried out using commercial kits and following the manufacturer's instructions.

### rtPA release from the CSM

Pharmacokinetic activity of rtPA were analyzed in healthy animals treated with (1) vehicle, (2) rtPA at 1 mg/kg as bolus, (3) CSM@rtPA (1 mg/kg of rtPA) as bolus; (4) CSM@rtPA/L (1 mg/kg of rtPA) as bolus. All treatments were administered as a final volume of 0.2 mL through the tail vein. For enzymatic analysis, blood samples were collected in a microtainer BD (Microtainer K2E Tubes. Ref: 365975, Franklin Lakes, NJ, USA) from the carotid artery in basal conditions (before treatment administration) and 1, 5, 15 and 40 min after the treatment administration. Blood samples were processed to obtain plasma, which was subsequently assayed using a commercial kit (Sensolyte AMC t-PA Activity Assay, Anaspec, France).

### Tail bleeding assay

The tail bleeding assay was performed in healthy animals treated with (1) vehicle, (2) rtPA at 1 mg/kg as bolus, (3) CSM@rtPA (1 mg/kg of rtPA) as bolus. After the administration of the treatments, 20 mm of the tail was cut using a blade. Immediately, the tail was inserted in an Eppendorf with 1 mL of saline pre-heated to 37 °C, where the blood was collected. For 20 min bleeding time and re-bleedings were controlled.

### Thromboembolic model

Thromboembolic stroke model was induced by injection in the MCA of mice as originally described Orset et al. [28] Mice were placed in a stereotaxic frame, the skin between the right ear and eye was cut, the temporal muscle was retracted, and the temporal and parietal bones exposed. A small craniotomy was performed over the artery bifurcation, the meninges were cut using a 25 G needle (BD Microlance, Ref. 300600, Italy) and the MCA was exposed.

A micropipette (tip size: 20–40 µm), made with hematologic glass capillaries (World Precision Instruments, Florida, USA) using a puller (Sutter Instruments, California, USA), was pneumatically filled with 1.5 µL of 1.5 U/µL thrombin (Murine Thrombin 0.05 mg MIIA. Stago-BNL, Belgium). The micropipette was placed in a micromanipulator and 1 μl of thrombin solution was injected into the lumen of the artery bifurcation to induce the formation of a clot. The micropipette was removed 15 min later, when the clot was stabilized.

CBF was monitored with a Periflux 5000 laser Doppler perfusion monitor (Perimed AB, Sweden) by placing the Doppler probe (MT B500-0L240, Straight Microtip, Perimed, Sweden) in the parietal territory of the MCA. Basal CBF and throughout the experiment was measured. Artery occlusion was considered successful when the CBF downfall was more than 60% relative to the basal.

After 30 min, the different treatments were administered intravenously into the tail vein. A small incision was made in the animal´s tail. The skin was cut out, and the tail vein was exposed. A 30 G needle (BD Microlance, Ref. 4656300) was used for every treatment administration; the puncture is rapidly closed to prevent bleeding.

The groups of treatment (n = 8) were the following: (1) vehicle (saline); (2) 1 mg/kg of rtPA administered 10% as bolus and 90% as infusion, that is the clinical dose; (3) 1 mg/kg of rtPA as bolus; 4) 1 mg/kg of rtPA-FITC as bolus; (5) CSMs without rtPA (corresponding to the same number of CSM@rtPA) as bolus (6) CSM@rtPA (1 mg/kg of rtPA) as bolus; (7) CSM@rtPA/L (1 mg/kg of rtPA) as bolus.

### Magnetic resonance imaging analysis

MRI studies were conducted on a 9.4 T horizontal bore magnet (BrukerBioSpin, Ettligen, Germany) with 12 cm wide actively shielded gradient coils (440 mT/m). Radiofrequency transmission was achieved with a birdcage volume resonator; signal was detected using a two-element arrayed surface coil (RAPID Biomedical, Germany), positioned over the head of the animal, which was fixed with a teeth bar, earplugs and adhesive tape. Respiratory frequency and body temperature were monitored throughout the experiment. Transmission and reception coils were actively decoupled from each other. Gradient-echo pilot scans were performed at the beginning of each imaging session for accurate positioning of the animal inside the magnet bore.

The progression of ischemic lesions and infarct volumes were determined from T2-maps calculated from T2-weighted images. The ischemic lesion was determined by counting pixels with apparent T2-map values above a threshold in the ipsilateral brain hemisphere.

In healthy mice, the T2-map values of the brain are over 50 ms. In the ipsilateral ischemic hemisphere, hyperintensity on T2-map determined the analysis of the ischemic damage (T2 map values > 60 ms).

T2-weighted images were acquired using a multi-slice multi-echo (MSME) sequence with a 11 ms echo time (TE), 2.8 s repetition time (TR), 12 echoes with 11 ms echo spacing, FA of 180º, 2 averages, 50 kHz spectral bandwidth (SW), 16 slices of 0.5 mm, 19.2 × 19.2 mm^2^ FOV with saturation bands to suppress signal outside this FOV, a matrix size of 256 × 256 (isotropic in-plane resolution of 75 μm/pixel × 75 μm/pixel) and implemented without fat suppression option. The acquisition time was 23 min.

T2*-weighted images were acquired using a multi-gradient-echo sequence (MGE) with a 5 ms TE, 1.2 s TR, 8 echoes with 4.5 ms echo spacing, 100 kHz spectral bandwidth, FA of 20º, 16 slices of 0.55 mm, 2 averages, 19.2 × 19.2 mm^2^ FOV with saturation bands to suppress signal outside this FOV, a matrix size of 256 × 256 (isotropic in-plane resolution of 75 μm/pixel × 75 μm/pixel) and implemented with fat suppression option. The acquisition time was 10 min.

### Statistical analysis

All data are presented as the mean and standard error of the mean (SEM) (mean ± SEM). Data were first examined to assess the distribution using the Kolmogorov–Smirnov normality test. Statistical analysis was assessed by Kruskal–Wallis test followed by Dunn’s multiple comparison in non-parametric data. One-way analysis of variance (ANOVA) followed by Dunnet’s or Tukey’s multiple comparison test was used to detect significant differences in parametric data. Statistical significance was set at P < 0.05. Statistical analysis and graph representations were performed using GraphPad Prism 8.0.

### Supplementary Information

**Additional file 1: Figure S1:** DLS graphs (in number) of CSM, CSM@rtPA and lyophilized CSM@rtPA**. Table S1:** Mean average hydrodynamic diameter and PDI values obtained by DLS measurements. **Figure S2:** NTA graphs of CSM, CSM@rtPA and lyophilized CSM@rtPA. **Table S2:** Mean average size and concentration values obtained by NTA measurements. **Figure S3:** SEM micrographs of the samples after uranyl acetate staining. **Figure S4:** Calibration curves of protein concentration (Bradford assay) and rtPA labeled with FITC. **Figure S5:** CSM and CSM@rtPA dispersions analysis by flow cytometry. **Figure S6:** Surface characterization of CD42 and CD47 markers in platelet and CSM samples by flow cytometry**. Figure S7:** proteolytic activity of free and encapsulated rtPA determined by a chromogenic substrate**. Figure S8**: In vitro cytotoxicity of CSM and CSM@rtPA.

## Data Availability

Data sharing is applicable to this article.

## Abbreviations

CBF: Cerebral blood flow
CFS: Cerebrospinal
CSM: Cellsome
DLS: Dynamic light scattering
FC: Flow cytometry
HT: Hemorrhagic transformation
IL: Interleukin
MFI: Mean fluorescence intensity
MRI: Magnetic resonance image
NTA: Nanoparticle tracking analysis
PBS: Phosphate buffer saline
RBC: Red blood cells
