# Supplementary material for: Thrombolytic therapy based on lyophilized platelet-derived nanocarriers for ischemic stroke
Source: J Nanobiotechnology. 2024 Jan 3;22:10. doi: 10.1186/s12951-023-02206-5 (PMC10763438; doi:10.1186/s12951-023-02206-5)
Supplement: Supplementary file 1 — Additional file 1: Figure S1: DLS graphs (in number) of CSM, CSM@rtPA and lyophilized CSM@rtPA. Table S1: Mean average hydrodynamic diameter and PDI values obtained by DLS measurements. Figure S2: NTA graphs of CSM, CSM@rtPA and lyophilized CSM@rtPA. Table S2: Mean average size and concentration values obtained by NTA measurements. Figure S3: SEM micrographs of the samples after uranyl acetate staining. Figure S4: Calibration curves of protein concentration (Bradford assay) and rtPA labeled with FITC. Figure S5: CSM and CSM@rtPA dispersions analysis by flow cytometry. Figure S6: Surface characterization of CD42 and CD47 markers in platelet and CSM samples by flow cytometry. Figure S7: proteolytic activity of free and encapsulated rtPA determined by a chromogenic substrate. Figure S8: In vitro cytotoxicity of CSM and CSM@rtPA. [file 12951_2023_2206_MOESM1_ESM.docx]

**Additional file 1**


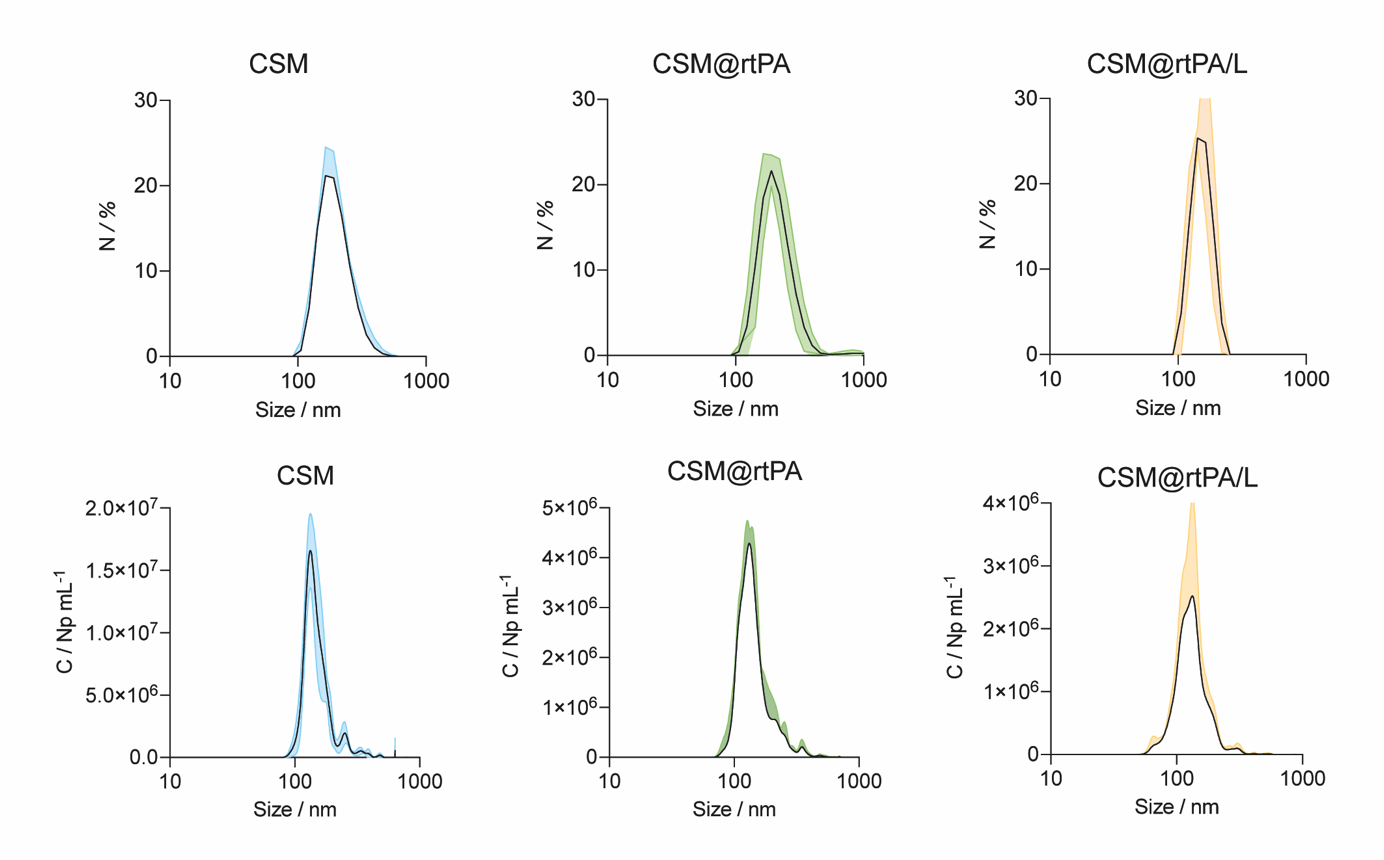


**Figure S1**. DLS size distribution of CSM, CSM@rtPA and lyophilized CSM@rtPA. Data are shown as mean ± SEM. (n=3 independent samples). DLS was used to characterize the colloidal properties of the CSMs and determine the hydrodynamic size of the samples.

**Table S1**. Size distribution analysis by DLS of CSM, CSM@rtPA and lyophilized CSM@rtPA. Mean hydrodynamic diameter derived from the DLS size distributions of the samples dispersed in PBS buffer. **^[1]^** d_H,I_, d_H,N_ refer to the mean average hydrodynamic diameter from the intensity, and number of DLS distributions, respectively. PDI refers to polydispersity index. **^[2]^** ζ-potential values of CSM, CSM@rtPA and lyophilized CSM@rtPA dispersed in Mili-Q water. Data are shown as mean ± SEM (n = 3 independent samples). Standard deviation values were calculated from three independent measurements of three independent CSMs samples.

| Sample | PDI | d_H,I_ (nm) ^[1]^ | d_H,N_ (nm) ^[1]^ | ζ-Potential (mV) ^[2]^ |
| --- | --- | --- | --- | --- |
| CSMs | 0.26 ± 0.032 | 206 ± 0.82 | 225 ± 1.4 | -15.93 ± 1.15 |
| CSM@rtPA | 0.325 ± 0.043 | 219.7 ± 10.5 | 234.5 ± 0.7 | -17.50 ± 1.51 |
| CSM@rtPA / L | 0.45 ± 0.07 | 258 ± 15.5 | 261.2 ± 16 | -16.4 ± 0.92 |


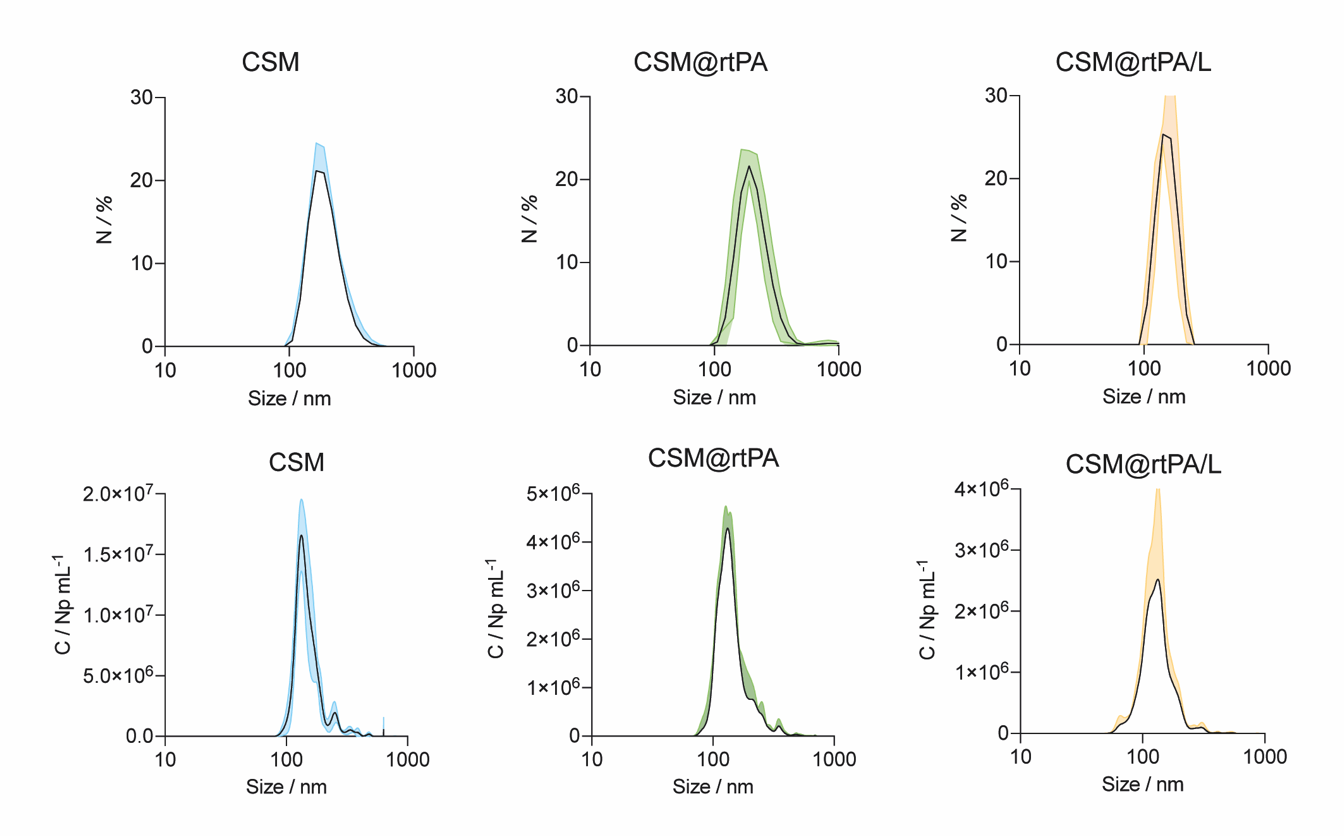


**Figure S2**. NTA size distribution of CSM, CSM@rtPA and lyophilized CSM@rtPA. Data are shown as mean ± SEM (n=3 independent samples). NTA was used to characterize the colloidal properties of the CSMs and determine the CSMs concentration in solution.

**Table S2**. Raw data of the mean hydrodynamic diameter for the NTA distributions and concentration of three independent measurements of CSM, CSM@rtPA and lyophilized CSM@rtPA. Data are shown as mean ± SEM. (n = 3 independent samples).

| Sample | Size (nm) | Concentration  (CSM/mL) |
| --- | --- | --- |
| CSMs | 182.8 ± 3.1 | (2.70 ± 1.3) ·10^11^ |
| CSM@rtPA | 184.4 ± 5.8 | (2.65 ± 0.7) ·10^11^ |
| CSM@rtPA / L | 187.3 ± 3.0 | (1.23 ± 0.2) ·10^11^ |


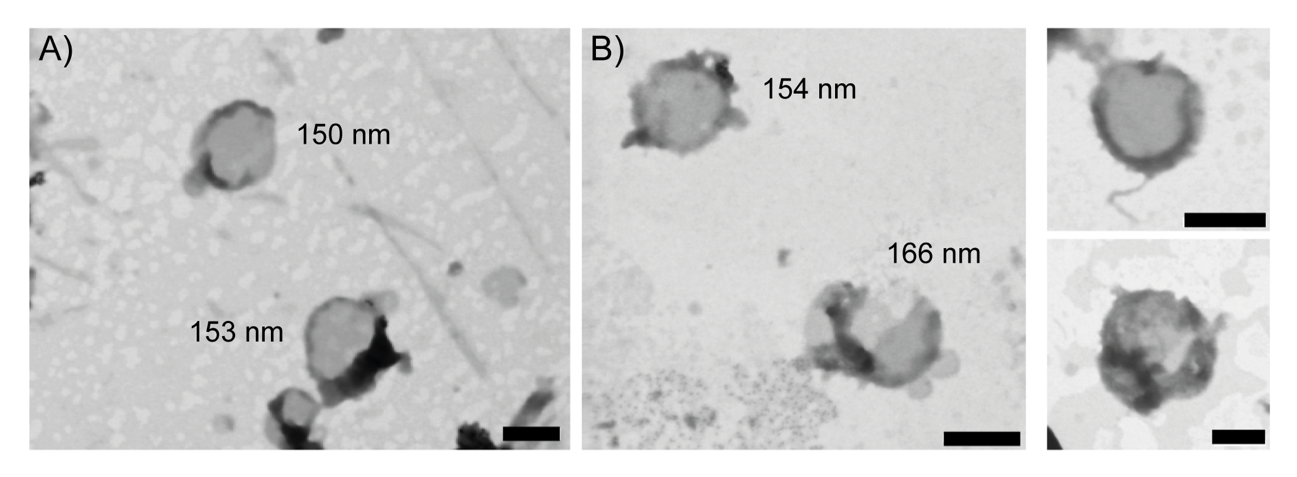


**Figure S3**. STEM-in SEM micrographs of CSMs staining with uranyl acetate. A) CSM samples. B) CSM@rtPA samples. Scale bars: 200 nm


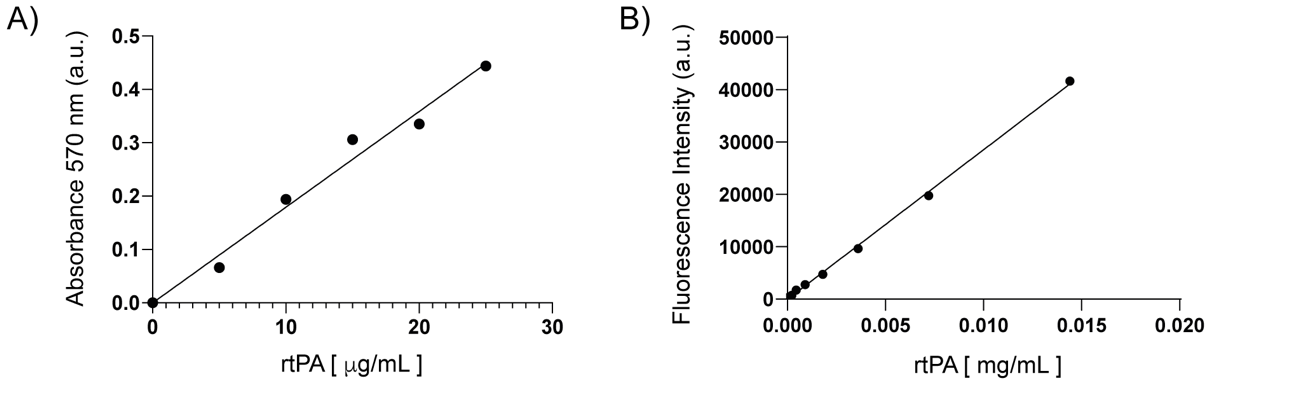


| Sample | [rtPA] mg/mL | % LE |
| --- | --- | --- |
| CSM@rtPA | 0.110 ± 0.023 | 21.7 ± 1 |
| CSM@rtPA / L | 0.103 ± 0.025 | 19.2 ± 4.6 |

**Figure S4**. Calibration curves. A) Protein quantification by Bradford assay: absorbance at 570 nm versus protein concentration (y = 0.0187x – 0.0144, r^2^= 0.98). B) Fluorescence intensity of FTIC-rtPA versus rtPA concentration (y = 3•10^6^x – 150, r^2^ = 0.99). The concentration of rtPA encapsulated in 1mL of CSM sample was around 0.11 ± 0.02 mg rtPA/mL. Once we determine the concentration of rtPA encapsulated in the CSM sample and the concentration of CSM analyzed by NTA, the number of rtPA molecules per CMS nanoparticle was estimated around 4000 rtPA/CMS. The % of loading efficiency (LE) was calculated by dividing the quantity of the cargo obtained from the encapsulation process by the initial quantity of cargo (0.4 mg of rTPA) added to 1mL of CSMs solution. The % of LE remains constant among the different batches synthesized (21.7 ± 1 %).


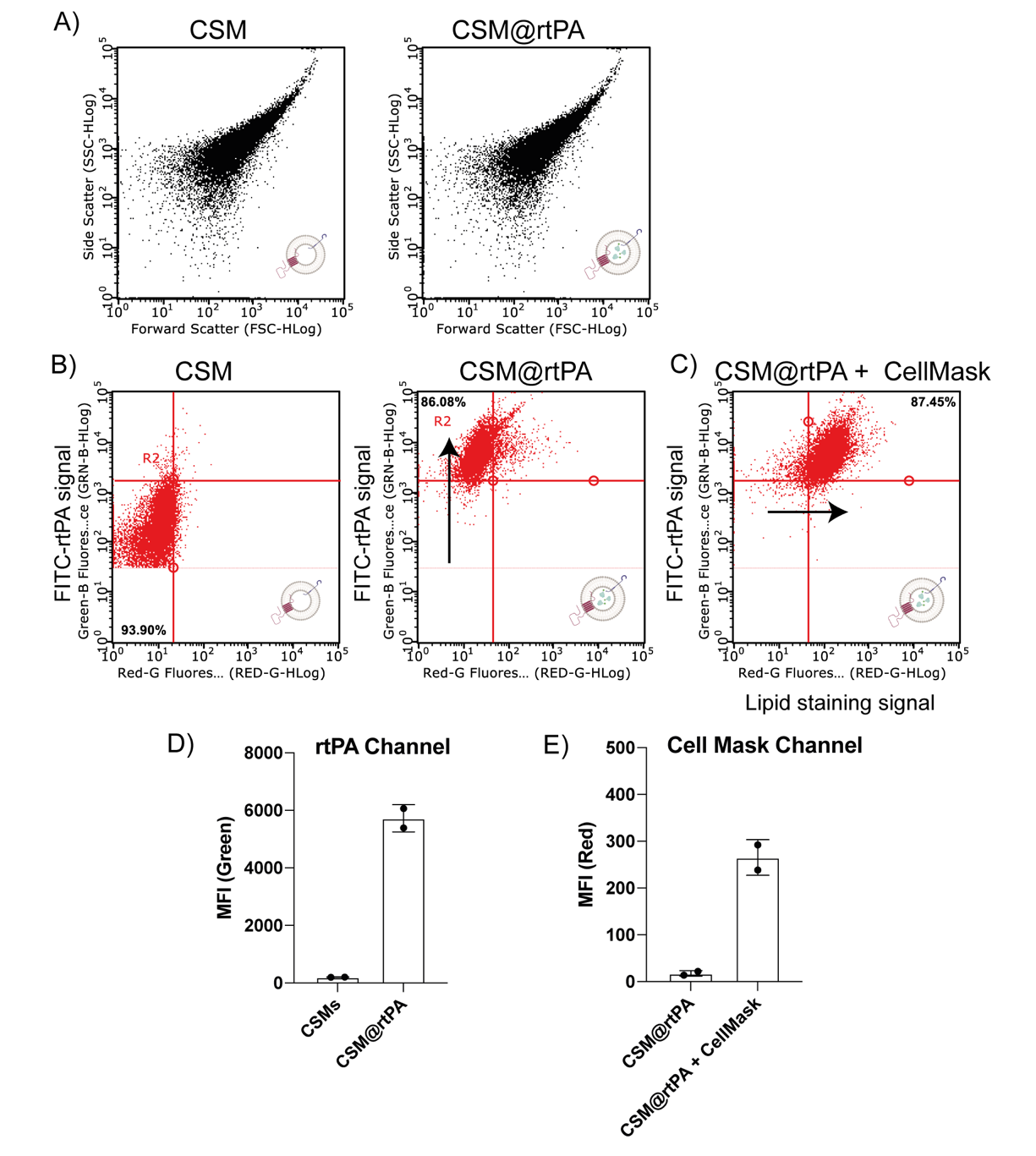


**Figure S5**. CSM dispersion analysis by flow cytometry. The variation of the flow cytometry side scattering signal and the fluorescence signals of CSM and CSM@rtPA samples are shown. A) Scatter density plots of side scattering (SSC) signal versus forward scattering (FSC) signal for CSM and CSM@rtPA samples. B) Scatter density plots of Green fluorescence signal (Green-B channel, rtPA channel) versus Red fluorescence signal (Red-R channel, CellMask DeepRed channel) for CSM and CSM@rtPA samples without any label. C) Scatter density plots of Green fluorescence signal (Green-B channel, rtPA channel) versus Red fluorescence signal (Red-R channel, CellMask DeepRed channel) for CSM@rtPA sample after labeling with CellMask Deep Red for lipid staining.

*
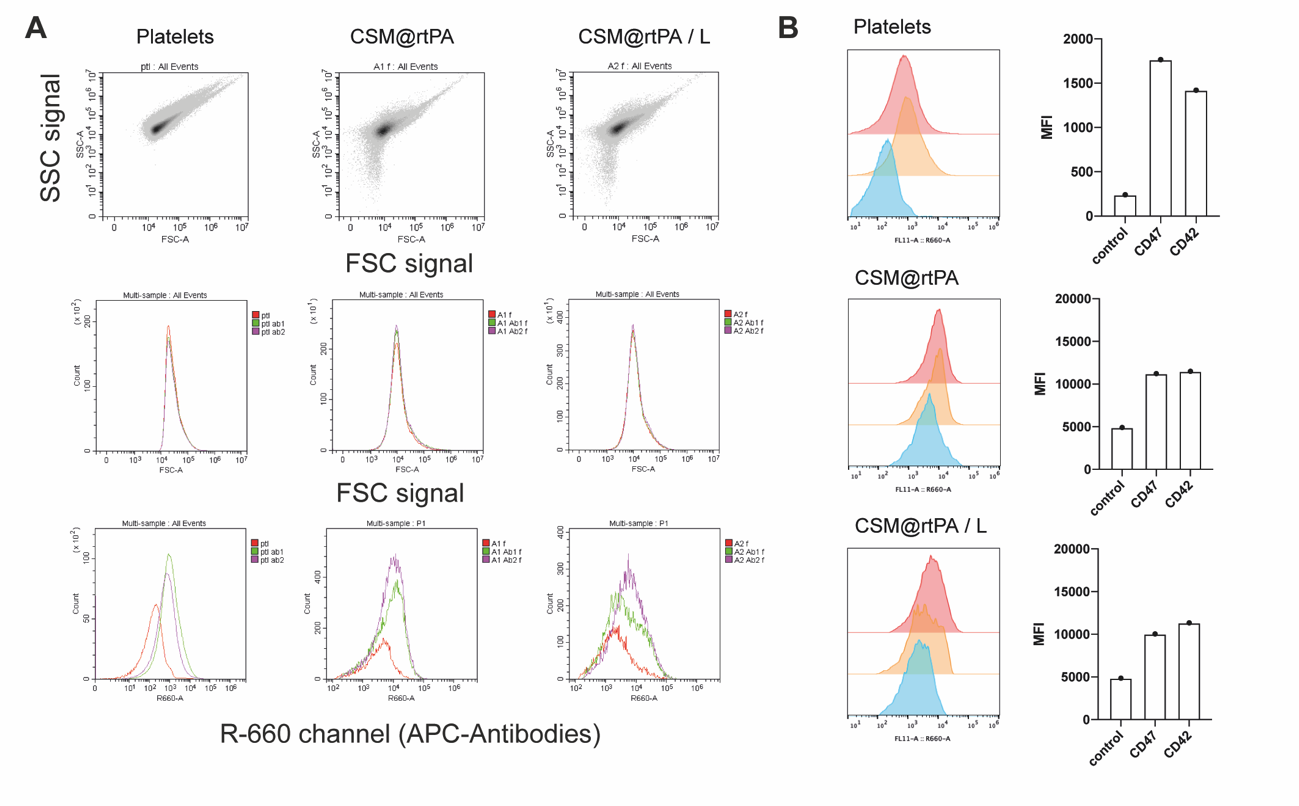
*

**Figure S6**. Surface characterization of CD42 and CD47 in platelet and CSM@rtPA after synthesis and after lyophilization (CSM@rtPA/L) process by Flow cytometer. A) Scatter density plots of side scattering (SSC) signal versus forward scattering (FSC) signal for platelets, CSM@rtPA amd CSM@rtPA/L samples. Histograms representation of mean FSC and mean fluorescence intensity (MFI) of Red-R-channel (R-660) of platelets, CMS@rtPA, and CSM@rtPA/L samples before and after antibody labeling. B) Overlay representation of MFI of R-660 channel of samples control (in blue) and samples treated with APC-fluorescently labeled anti-hCD47 antibody (in orange), and APC-fluorescently labeled anti-hCD42b/GPlbα antibody (in red).


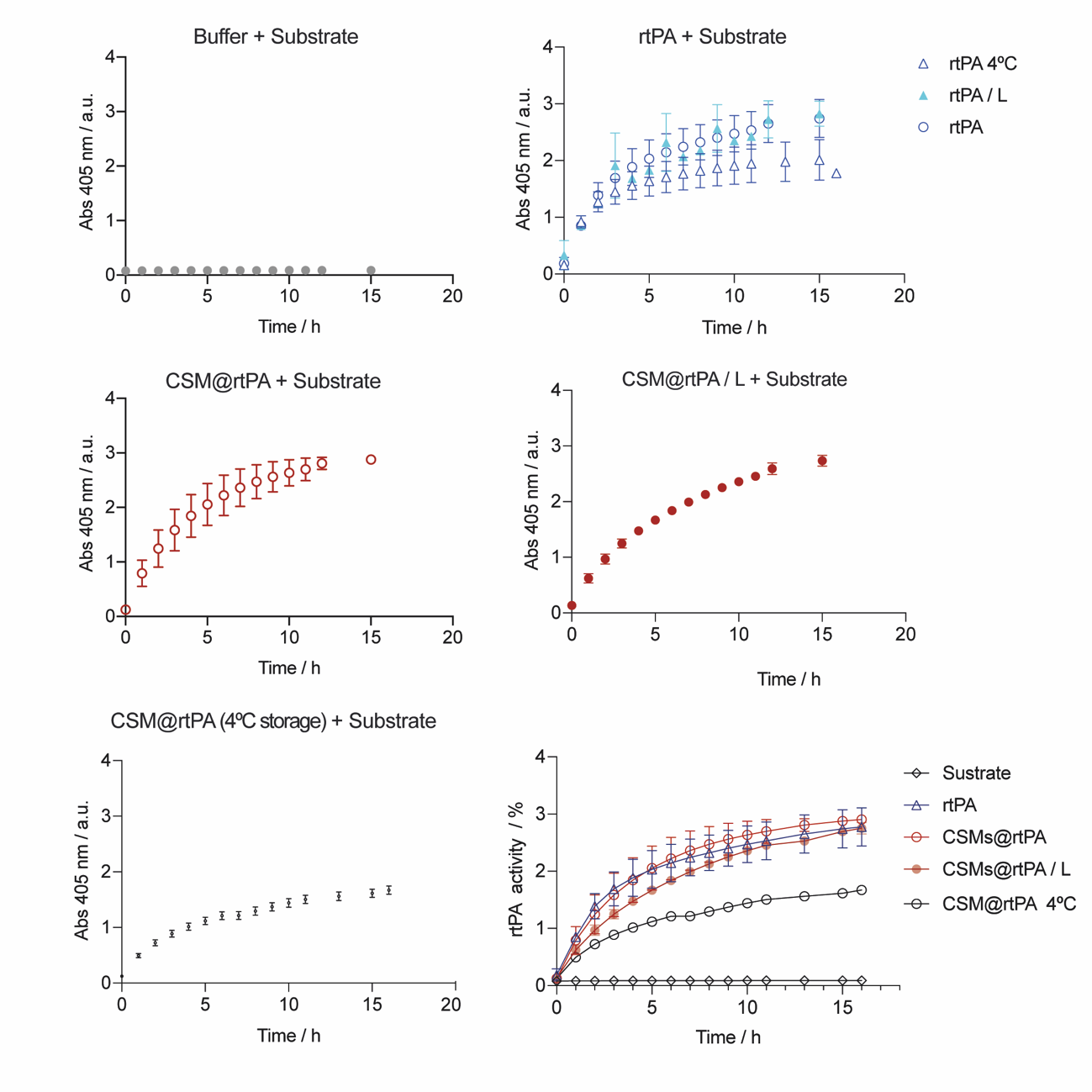


**Figure S7**. rtPA bioactivity studies. The proteolytic activity of free rtPA (and free rtPA storage at 4ºC or after lyophilization process for several days) and encapsulated rtPA (CSM@rtPA), before and after lyophilization process, in presence of the chromogenic substrate is recorded by measuring the absorbance at 405 nm over time. These data represent mean ± S.D. (n=3 independent replicates).


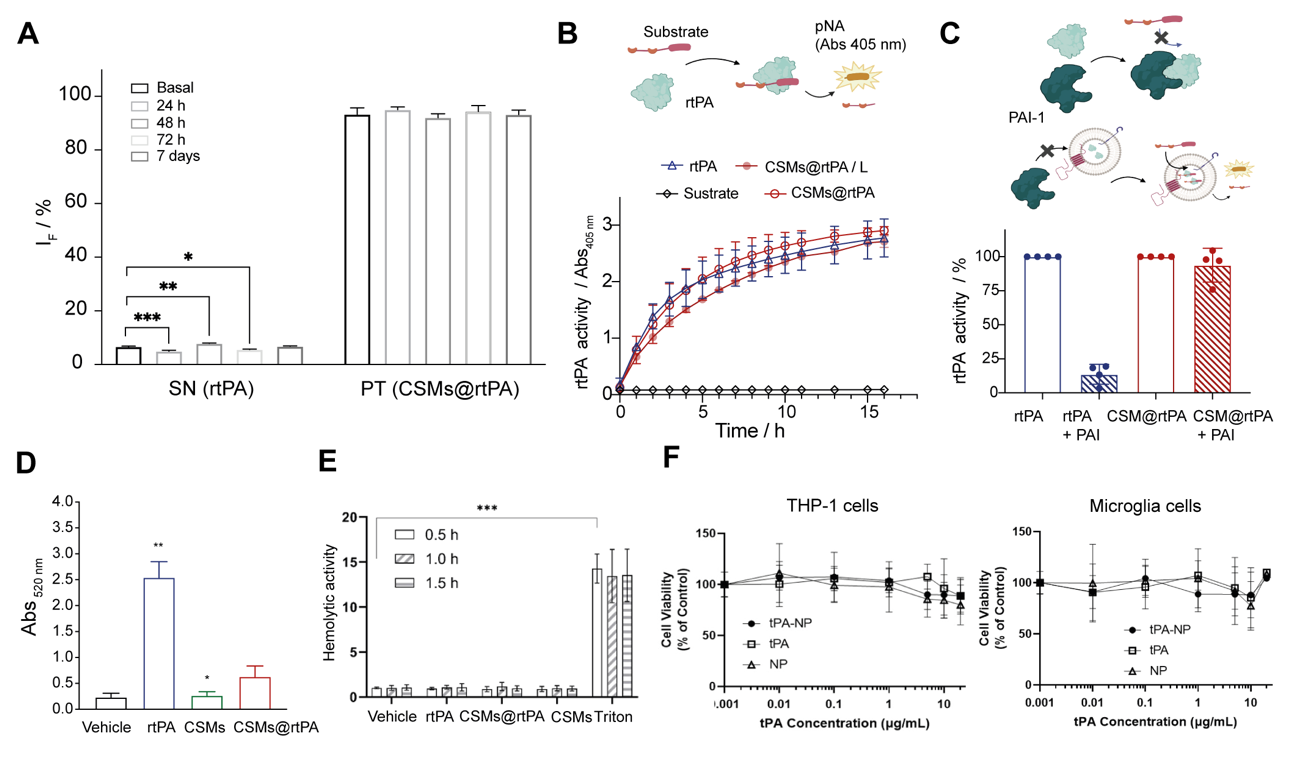


**Figure S8**. THP-1 macrophage and HMC3 microglia cells were treated for 24 hours with increasing concentrations of rtPA, CSM, CSM@rtPA/L. Cell nuclei were stained with Hoechst 33342 nucleic acid staining and counted using the Cell Counter plugin in FIJI (ImageJ). Cell counts normalized to the mean cell count of untreated cells. Data represents means ± S.D. (n=3 independent experiments.
